# Comparing the interobserver reproducibility of different regions of interest on multi-parametric renal magnetic resonance imaging in healthy volunteers, patients with heart failure and renal transplant recipients

**DOI:** 10.1007/s10334-019-00809-4

**Published:** 2019-12-10

**Authors:** Alastair J. Rankin, Sarah Allwood-Spiers, Matthew M. Y. Lee, Luke Zhu, Rosemary Woodward, Bernd Kuehn, Aleksandra Radjenovic, Naveed Sattar, Giles Roditi, Patrick B. Mark, Keith A. Gillis

**Affiliations:** 1 Room 311, Institute of Cardiovascular and Medical Sciences, BHF Glasgow Cardiovascular Research Centre, University of Glasgow, 126 University Place, Glasgow, G12 8TA UK; 2 Department of Clinical Physics and Bioengineering, NHS Greater Glasgow and Clyde, Glasgow, UK; 3 Clinical Research Imaging, NHS Greater Glasgow and Clyde, Glasgow, UK; 4 Siemens Healthcare GmbH, Erlangen, Germany; 5 Department of Radiology, NHS Greater Glasgow and Clyde, Glasgow, UK

**Keywords:** Renal MRI, Reproducibility, Chronic kidney disease, Renal transplantation, Heart failure

## Abstract

**Objective:**

To assess interobserver reproducibility of different regions of interest (ROIs) on multi-parametric renal MRI using commercially available software.

**Materials and methods:**

Healthy volunteers (HV), patients with heart failure (HF) and renal transplant recipients (Tx) were recruited. Localiser scans, T1 mapping and pseudo-continuous arterial spin labelling (pCASL) were performed. HV and Tx also underwent diffusion-weighted imaging to allow calculation of apparent diffusion coefficient (ADC). For T1, pCASL and ADC, ROIs were drawn for whole kidney (WK), cortex (Cx), user-defined representative cortex (rep-Cx) and medulla. Intraclass correlation coefficient (ICC) and coefficient of variation (CoV) were assessed.

**Results:**

Forty participants were included (10 HV, 10 HF and 20 Tx). The ICC for renal volume was 0.97 and CoV 6.5%. For T1 and ADC, WK, Cx, and rep-Cx were highly reproducible with ICC ≥ 0.76 and CoV < 5%. However, cortical pCASL results were more variable (ICC > 0.86, but CoV up to 14.2%). While reproducible, WK values were derived from a wide spread of data (ROI standard deviation 17% to 55% of the mean value for ADC and pCASL, respectively). Renal volume differed between groups (*p* < 0.001), while mean cortical T1 values were greater in Tx compared to HV (*p* = 0.009) and HF (*p* = 0.02). Medullary T1 values were also higher in Tx than HV (*p* = 0.03), while medullary pCASL values were significantly lower in Tx compared to HV and HF (*p* = 0.03 for both).

**Discussion:**

Kidney volume calculated by manually contouring a localiser scan was highly reproducible between observers and detected significant differences across patient groups. For T1, pCASL and ADC, Cx and rep-Cx ROIs are generally reproducible with advantages over WK values.

**Electronic supplementary material:**

The online version of this article (10.1007/s10334-019-00809-4) contains supplementary material, which is available to authorized users.

## Introduction

Functional renal imaging is a burgeoning field of research that has the potential to translate into meaningful clinical applications for patients with kidney disease [1]. Multi-parametric magnetic resonance imaging (MRI) allows acquisition of multiple sequences with potential to inform regarding structure, tissue composition, perfusion, and physiology of renal function in a single scan [2]. However, the clinical utility of each sequence, and indeed the potential additive benefit of their use together, are yet to be proven. The immediate research priority in renal MRI is focusing on the standardisation and harmonisation of image acquisition across research sites and MRI vendors. This ‘ground-up’ approach is driven by international, independently funded working groups, including PARENCHIMA [2], a subsidiary of the European Cooperation in Science and Technology (COST) Action group and the UK Renal Imaging Network (UKRIN), amongst others. As image acquisition is standardised, scientific scrutiny must also be applied to the methods of analysis. Many of the MRI sequences employed produce quantitative results from modelling dependent on measurements using other sequences [3], and for which the resultant values will vary depending on whether whole kidney, renal cortex or renal medulla is selected [4]. Numerous analytic approaches have been reported to date, and the optimal technique in terms of time and clinical relevance, are not yet known. In addition, the absence of commercially available analysis software that is specifically designed for unique interests of renal MRI leads to use of in-house bespoke software, which renders external validation of results challenging.

Our centre has an active renal MRI research group, with current projects exploring the clinical implications of multi-parametric renal MRI across healthy volunteers [5] as well as patients with heart failure, chronic kidney disease (CKD) [6] and renal transplants. We aim to compare different regions of interest (ROIs) and their interobserver reproducibility using commercially available analysis software in healthy and patient populations, including native and transplant kidneys, across selected MRI sequences.

## Methods

### Study population and clinical parameters

Patients were recruited from nephrology and cardiology clinics, and from general advertisement, for the renal transplant (Tx), heart failure (HF) and healthy volunteer (HV) cohorts, respectively. For Tx and HF patients, the scans were acquired as baseline imaging for two separate ongoing clinical studies (ClinicalTrials.gov: NCT03705091 and NCT03485092). Basic biometric parameters were measured and serum creatinine was measured in accredited clinical biochemical laboratories. Estimated glomerular filtration rate (eGFR) was derived using the Chronic Kidney Disease Epidemiology Collaboration (CKD-EPI) equation [7]. All participants gave written informed consent and regional ethics committee approval was granted; the study was conducted in agreement with the Declaration of Helsinki.

### MRI acquisition

**Fig. 1:**
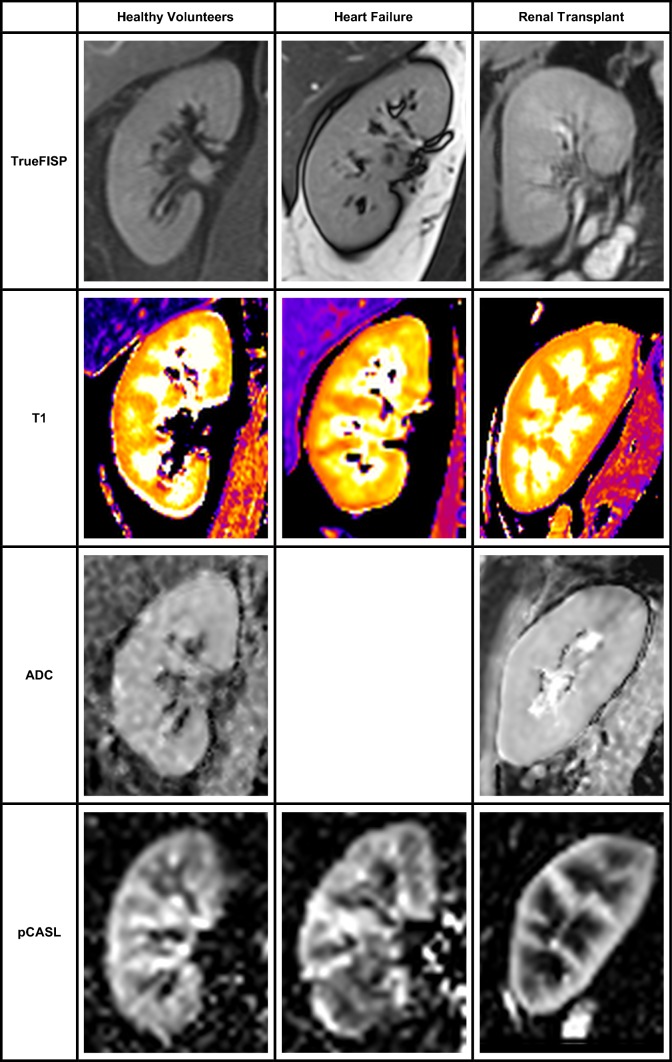
Representative image of each MRI sequence for each participant group

MRI was performed on a Siemens MAGNETOM Prisma 3T scanner (Siemens Healthcare, Erlangen, Germany) using an 18-channel phased array coil anteriorly and a 32-channel spine coil posteriorly. Scans for renal volume, perfusion and T1 were acquired from all patients (Fig. 1), with the transplant kidney scanned for the Tx group. Diffusion-weighted imaging (DWI) was performed on the Tx and HV cohorts. Patients were imaged supine.

- *Volume* Coronal images were acquired during a breath hold at expiration using a steady-state free precession sequence [true fast imaging with steady-state precession (TrueFISP)]. The imaging parameters used are listed in Supplementary Material Table 1 (HV and Tx cohorts) and Supplementary Material Table 2 (HF cohort).
- *T1* T1 maps were acquired for a single coronal oblique slice through the centre of the kidney using a modified look-locker inversion recovery (MOLLI) [8, 9] sequence with single shot TrueFISP readout [10]. For Tx and HV cohorts, images were acquired at 14 different inversion times (TI) [acquisition scheme 11(3)3] with an initial delay of 180 ms after the first inversion pulse and a delay of 260 ms, after the second inversion pulse. The interval between subsequent measurements was 550 ms, resulting in TIs of 180, 260, 730, 810, 1280, 1360, 1830, 2380, 2930, 3480, 4030, 4580, 5130, 5680 ms) and echo spacing of 3.04 ms. The acquisition time was 10 s. Images were acquired during free breathing.
  For the HF cohort, images were acquired at eight different inversion times [acquisition scheme 5(3)3] with a start TI of 100 ms, a TI increment of 80 ms (inversion times dependent on captured cardiac cycle), a reported TR of 280–340 ms, and echo spacing of 2.44 ms. Images were acquired during a breath hold. Other imaging parameters are given in Supplementary Materials Tables 1, 2.
  Motion correction and fitting of the T1 map was performed using a phase-sensitive inversion recovery reconstruction implemented in the vendor software (Siemens, VE11C, MyoMaps) [11].

- *Arterial spin labelling* A pseudo-continuous arterial spin labelling (pCASL) scan [12] with a 3D turbo gradient spin-echo (TGSE) readout which was acquired during free breathing [13]. The prototype sequence comprises a slice-selective presaturation pulse to suppress the signal from preceding excitations and a frequency-offset-corrected inversion (FOCI) pulse positioned over the imaging region. This is followed by the pCASL slice-selective labelling pulse. For background saturation, four non-selective hyperbolic secant pulses are applied, interspersed with three slice-selective saturation pulses, positioned superior to the labelling plane.
  The pCASL labelling plane was positioned in a transverse oblique slice of thickness 10 mm perpendicular to the aorta and superior to the kidneys to label the blood in the descending aorta (Supplementary Material Fig. 1). The start time of the pCASL labelling was 3000 ms and the pCASL duration was 1500 ms with a flip angle of 28°. The presaturation pulses and FOCI pulse were positioned in a transverse slab covering the kidneys. The pulses to suppress inflowing arterial blood were applied in a slab superior to the labelling plane to suppress inflowing arterial blood. Images were obtained in a coronal oblique orientation covering the whole kidney volume. A low-resolution pCASL scan with one measurement was acquired to confirm that the positioning of the labelling plane was appropriate to produce signal in the perfusion-weighted image. This was followed by a higher resolution scan with parameters as given in Tables 1, 2. The sequence acquires label and control images and a reference proton density-weighted (M0) image.
  Perfusion maps were produced using inline software. In-plane 2D motion correction is applied, retrospectively, to proton density-weighted (*M*_0_) label and control images. Label and control images are subtracted to create perfusion-weighted images. Maps of perfusion rate (*f*) are calculated pixel by pixel using the motion-corrected proton density-weighted (*M*_0_) and perfusion-weighted (Δ*M*) images according to:
  $$\Delta M = f\frac{{2M_{0} }}{\lambda }T_{{1^{\prime } }} \alpha \exp \left( {\frac{ - \Delta t}{{T_{{1{\text{blood}}}} }}} \right)\exp \left( {\frac{{ - \left( {t - \tau - \Delta t} \right)}}{{T_{{1^{\prime } }} }}} \right)\left( {1 - \exp \left( {\frac{ - \tau }{{T_{{1^{\prime } }} }}} \right)} \right),$$
  where *f* is the perfusion rate in ml/100 mg/min; *t* is the time between labelling and imaging (3000 ms); *τ* is the duration of labelling pulse (1500 ms); Δ*t* is the arterial transit time, assumed to be 750 ms; *α* is the labelling efficiency, assumed to be 0.98; *λ* is the blood-tissue water partition coefficient, assumed to be 0.9 ml/100 g; *T*_1blood_ is the longitudinal relaxation time of arterial blood; *T*_1′_ is the apparent longitudinal relaxation time of tissue. A fixed *T*_1blood_ =* T*_1′_ = 1250 ms was assumed in calculating the perfusion maps.
  - *DWI* For the Tx and HV cohorts, DWI was performed using a single-shot spin-echo echo-planar imaging sequence with 17 slices positioned in a coronal oblique plane. Images were acquired at 10 *b* values (0, 50, 100, 150, 200, 250, 300, 500, 750, 1000 s/mm^2^) for four diffusion directions, averaged to give a 4-scan trace. Spectral attenuated inversion recovery (SPAIR) fat suppression was used and images were acquired during free breathing, with an acquisition time of 1 min 46 s. Apparent diffusion coefficient (ADC) maps were created using the vendor software, performing a mono-exponential fit to the ten *b*-values [14].

### MRI analysis

**Fig. 2:**
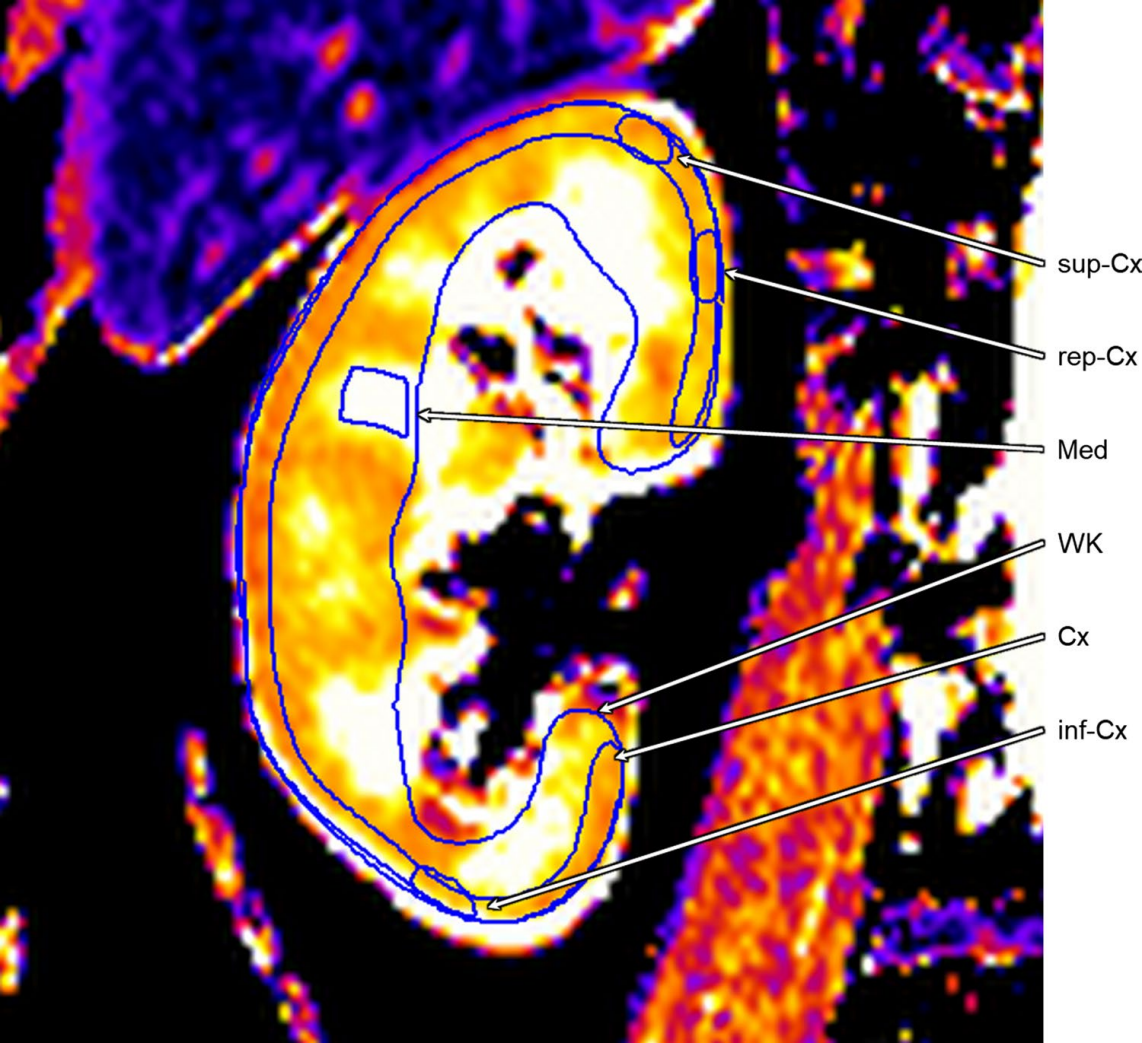
Representative image (T1) displaying the regions of interest drawn for whole kidney (WK), cortex (Cx), representative cortex (rep-Cx), superior cortex (sup-Cx), inferior cortex (inf-Cx) and medulla (Med)

Interobserver variability was compared across different methods of image analysis. For kidney volume, the renal contours were drawn around the whole kidney (excluding the renal pelvis) on the first and last slices containing renal tissue. Contours were then added to every alternate slice in between. This initial total kidney volume (linear interpolation for non-contoured slices) was then recorded (‘alternate slices’) prior to drawing contours to the remaining slices and noting the resultant volume (‘every slice’). For pCASL and DWI, a single slice was chosen for analysis. ROIs were drawn manually around the whole kidney (WK), cortex (Cx), an area of user-defined representative cortex (rep-Cx), within the cortex at the superior and inferior poles (sup-Cx and inf-Cx, respectively) and in a representative area of medulla (Med) (Fig. 2). Corticomedullary differentiation was assessed by ratio of Cx to Med. Each cohort was analysed by a pair of independent observers from a pool of four clinicians and one physicist, all with local training in renal MRI analysis (SAS and LZ analysed HV, SAS and MMYL analysed HF and KAG and AJR analysed Tx). Image analysis was performed using the commercially available software cvi42 version 5.9.4 (Circle Cardiovascular Imaging, Calgary, Canada).

### Statistical analysis

Descriptive statistics are reported as mean and standard deviation or median and range/interquartile range (IQR) for normally distributed and skewed data, respectively. Paired t-tests were used to compare kidney volume techniques and results were displayed graphically using a Bland–Altman plot [15]. Pearson correlation coefficient was used to quantify linear relationships between continuous variables. A total of 12 participants are required to detect a correlation coefficient of 0.8 with 90% power and alpha 0.05. Our decision to include 40 participants yields a power > 99.9% to detect a correlation coefficient of 0.8 at alpha 0.05. Interobserver reproducibility was measured using coefficient of variation (CoV) (calculated by the standard deviation divided by the mean) and intraclass correlation coefficient (ICC) (two-way random, average measures). One-way ANOVA was used to compare mean results across the three participant groups, with t-tests to interrogate pairs where groups differed. The mean value of the two observers is reported unless otherwise stated. All analyses were performed using SPSS Statistics Version 25.0 (Armonk, NY: IBM Corp.) and a conventional significance level of < 0.05 was used. Figures were generated using SPSS Statistics Version 25.0 (Armonk, NY: IBM Corp.) and Microsoft PowerPoint^®^ 2019.

## Results

### Participant demographics

A total of 40 participants were included: ten healthy volunteers, ten patients with heart failure (with reduced ejection fraction of ≤ 40%) and 20 renal transplant recipients. Clinical characteristics are shown in Table 1.

**Table 1:** Patient demographics and clinical characteristics

	All (*n* = 40)	HV (*n* = 10)	HF (*n* = 10)	Tx (*n* = 20)
Age (years), median (IQR)	56 (39–63)	43 (30–58)	62 (54–70)	51 (38–61)
Male (*n*, %)	28 (70%)	4 (40%)	7 (70%)	17 (85%)
eGFR (ml/min/1.73 m^2^), median (IQR)	60.0 (37.7–76.7)	NA	77.1 (65.8–86.9)	48.4 (36.1–64.3)

*HV* healthy volunteers, *HF* heart failure, *Tx* renal transplant, *eGFR* estimated glomerular filtration rate, *IQR* interquartile range

### Renal volume

**Fig. 3:**
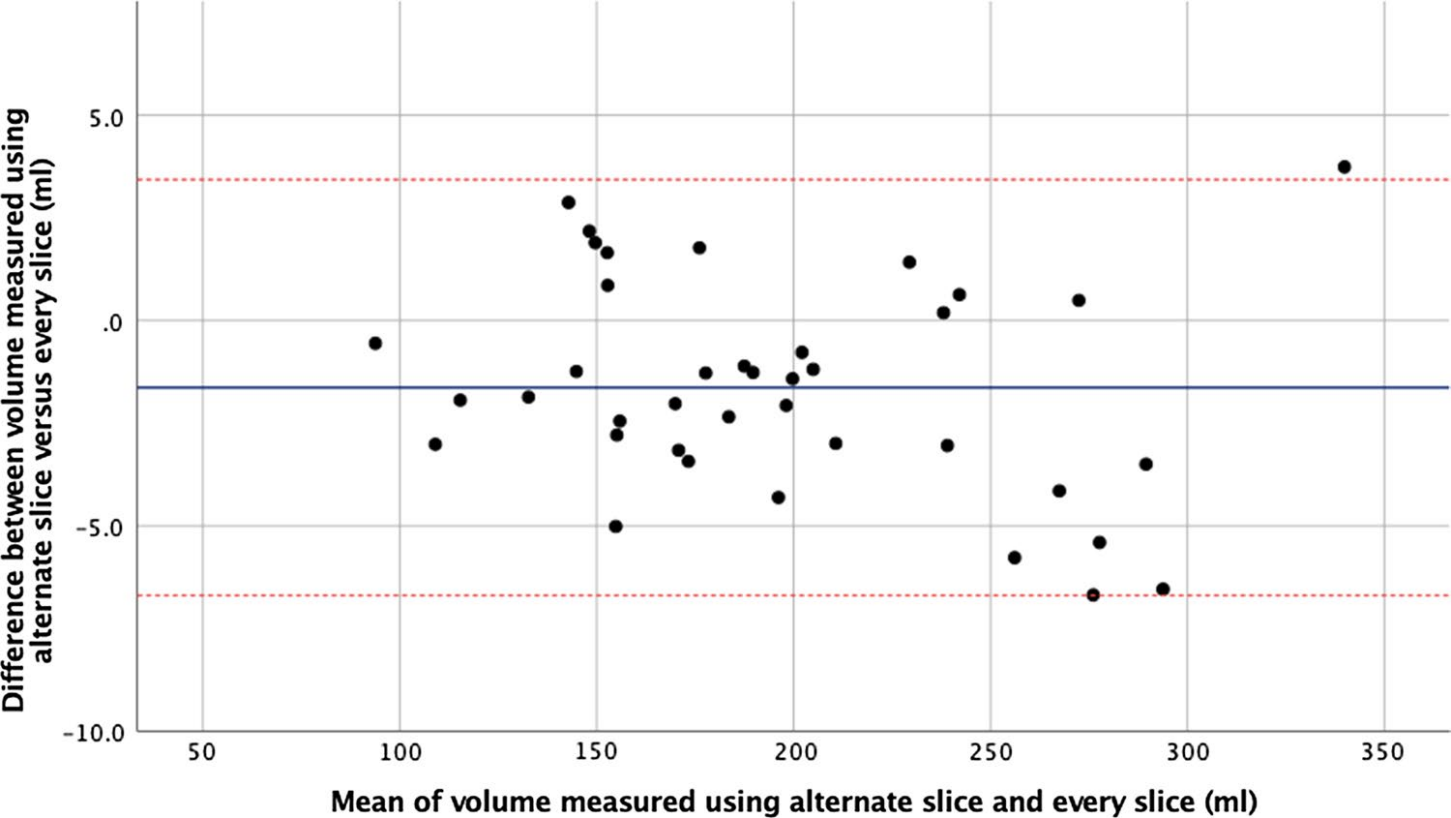
Bland–Altman plot comparing kidney volume as measured by contouring alternate slice versus every slice

Calculation of renal volume was possible in 39 patients (98%) (one patient did not have appropriate TrueFISP images). Mean difference in renal volume was 1.6 ml lower when contours were drawn on alternate slices as opposed to every slice (*p* < 0.001) (Fig. 3). There was no interobserver difference in renal volume with either approach (*p* = 0.56 for alternate slice, and *p* = 0.89 for every slice). Tables 2, 3 show the results and interobserver reproducibility for renal volume, respectively.

**Table 2:** Comparison of results depending on region of interest, MRI sequence and participant group

	All (*n* = 40)		HV (*n* = 20)		HF (*n* = 10)		Tx (*n* = 10)	
	Mean	SD	Mean	SD	Mean	SD	Mean	SD
**Volume (ml) (*n* = 39)**								
Alternate slice	195.8	56.8	147.8	30.9	170.6	45.3	230.1	49.2
Every slice	197.5	57.4	149.0	31.3	170.8	44.9	232.6	49.3
**T1 (ms) (*n* = 39)**								
Whole kidney	1772.8	131.4	1702.4	76.7	1696.5	85.7	1842.6	134.4
Cortex	1630.2	102.0	1557.7	104.1	1595.5	80.1	1680.1	86.3
rep-Cx	1606.1	114.4	1545.4	113.2	1543.8	71.1	1664.6	105.0
sup-Cx	1655.6	119.5	1606.8	149.8	1600.0	93.4	1705.3	98.1
inf-Cx	1639.0	103.7	1587.0	94.3	1590.0	113.3	1687.0	82.0
Med	1975.8	74.9	1899.0	80.5	1940.2	71.3	2028.1	74.2
Cortex: Med	0.83		0.82		0.82		0.83	
**pCASL (ml/min/100 g) (*n* = 37)**								
Whole kidney	181.7	56.6	187.5	58.4	161.6	47.1	190.2	60.7
Cortex	221.0	80.0	235.1	79.3	175.7	59.5	239.3	84.3
rep-Cx	260.8	91.4	271.8	93.5	228.4	92.3	273.3	90.4
sup-Cx	196.4	75.9	230.5	77.9	160.4	54.2	197.4	79.6
inf-Cx	225.2	105.2	213.8	91.1	161.8	84.4	269.2	107.5
Med	95.8	41.8	108.8	45.1	121.5	31.5	73.0	45.9
Cortex: Med	2.3		2.2		1.4		3.3	
**ADC (× 10**^**−6**^** mm**^**2**^**/s) (*n* = 28)**								
Whole kidney	1687.6	115.6	1687.2	97.4	–	–	1687.7	125.8
Cortex	1678.1	111.4	1704.0	96.8	–	–	1665.8	118.1
rep-Cx	1696.9	117.7	1719.9	158.6	–	–	1686.0	96.1
sup-Cx	1686.3	144.2	1720.4	120.6	–	–	1670.1	154.5
inf-Cx	1696.4	115.2	1700.5	111.8	–	–	1694.4	119.7
Med	1671.9	82.5	1726.3	93.9	–	–	1646.1	77.2
Cortex: Med	1.0		1.0				1.0	

The standard deviation presented represents the spread of mean values. Volume measured by contouring alternate slices was similar to contouring every slice in all groups. Within each group, the values for whole kidney, cortical and medullary regions of interest were different for T1 and pCASL. In contrast, ADC values were similar for whole kidney, cortical and medullary regions of interest

*HV* healthy volunteers, *HF* heart failure, *Tx* renal transplant, *SD* standard deviation, *rep-Cx* area of representative cortex, *sup-Cx* area of representative cortex at superior pole, *inf-Cx* area of cortex at inferior pole, *Med* medulla, *pCASL* pseudo-continuous arterial spin labelling, *ADC* apparent diffusion coefficient

**Table 3:** Interobserver reproducibility by MRI sequence and analysis approach

	CoV (%)	ICC
**Volume (ml) (*n* = 39)**		
Alternate slice	6.5	0.97
Every slice	6.7	0.96
**T1 (ms) (*n* = 39)**		
Whole kidney	1.0	0.97
Cortex	1.2	0.97
rep-Cx	2.0	0.95
sup-Cx	3.2	0.96
inf-Cx	2.5	0.86
Med	2.6	0.87
**pCASL (ml/min/100 g) (*n* = 37)**		
Whole kidney	7.0	0.90
Cortex	10.3	0.93
rep-Cx	14.2	0.86
sup-Cx	19.1	0.69
inf-Cx	14.6	0.92
Med	29.6	0.73
**ADC (× 10**^**−6**^** mm**^**2**^**/s) (*n* = 28)**		
Whole kidney	2.0	0.90
Cortex	2.6	0.85
rep-Cx	3.7	0.76
sup-Cx	5.0	0.64
inf-Cx	3.8	0.62
Med	5.5	0.50

Whole kidney and cortical ROIs were highly reproducible in all sequences

*CoV* coefficient of variation, *ICC* intraclass correlation coefficient, *rep-Cx* area of representative cortex, *sup-Cx* area of representative cortex at superior pole, *inf-Cx* area of cortex at inferior pole, *Med* medulla, *pCASL* pseudo-continuous arterial spin labelling, *ADC* apparent diffusion coefficient

### T1, pCASL, ADC: comparison of different ROIs

T1, pCASL and ADC sequences were acquired in 39, 39 and 28 patients, respectively. Image quality was acceptable in all but two pCASL acquisitions in the Tx group who were excluded from further analysis. Table 2 shows the mean results for each sequence depending on whether ROIs were drawn for WK, Cx, rep-Cx, sup-Cx, inf-Cx and Med. The standard deviation in Table 2 represents the spread of mean values obtained. Table 3 shows the interobserver reproducibility for each ROI by sequence and participant group. For T1 and ADC, WK, Cx and rep-Cx were highly reproducible (ICC ≥ 0.76; CoV < 5%). For pCASL, Cx and rep-Cx were less readily reproducible (ICC > 0.86 but CoV up to 14.2%). The reproducibility of Med ROI was excellent for T1, but less good for pCASL and ADC (Table 3). Table 4 shows the spread of data within each ROI by reporting the mean ROI standard deviation as a proportion of the mean value. The spread of data from WK ROIs was higher than cortex-specific ROIs, even when the mean value for each was similar (Table 2).

**Table 4:** Table representing the spread of data from which the mean is calculated depending on region of interest and MRI sequence

	ROI SD (mean)	ROI SD as proportion of mean value (%)
**T1 (ms) (*n* = 39)**		
Whole kidney	354.1	20.0
Cortex	125.7	7.7
rep-Cx	49.6	3.1
sup-Cx	71.4	4.3
inf-Cx	69.8	4.3
Med	74.9	3.8
**pCASL (ml/100 g/min) (*n* = 37)**		
Whole kidney	100.7	55.4
Cortex	85.1	38.5
rep-Cx	41.2	15.8
sup-Cx	48.0	24.5
inf-Cx	53.2	23.6
Med	41.8	43.6
**ADC (× 10**^**−6**^** mm**^**2**^**/s) (*n* = 28)**		
Whole kidney	289.5	17.2
Cortex	169.6	10.1
rep-Cx	71.5	4.2
sup-Cx	105.8	6.3
inf-Cx	85.9	5.1
Med	84.7	5.1

The ROI standard deviation is generated by the analysis software to represent the spread of values within each ROI. This table reports the mean ROI standard deviation for each sequence and displays it as a proportion of the mean value. The spread of data is larger for whole-kidney values, which includes cortical and medullary values as well as potential confounding data from vessels and renal pelvis. Conversely, the spread of data from the smaller ROIs of representative cortex may be uncharacteristically low if too small a ROI is drawn to be truly representative

*ROI* region of interest, *SD* standard deviation, *rep-Cx* area of representative cortex, *sup-Cx* area of representative cortex at superior pole, *inf-Cx* area of cortex at inferior pole, *Med* medulla, *pCASL* pseudo-continuous arterial spin labelling, *ADC* apparent diffusion coefficient

### Correlation between different ROIs

For T1, the correlation coefficient for Cx compared to WK, rep-Cx, sup-Cx, inf-Cx and Med was 0.76, 0.93, 0.86, 0.85 and 0.62, respectively. The corresponding values for pCASL were 0.92, 0.91, 0.84, 0.81 and 0.26; and for ADC 0.87, 0.79, 0.75, 0.85 and 0.78 [*p* < 0.001 for all, except pCASL Med which was not significant (*p* = 0.13)].

### Comparison between participant groups

There was a significant difference in kidney volume between groups (*F* = 13.2, *p* < 0.001) with the greatest renal volume in Tx, then HF and then HV (Table 2). Mean T1 values also differed between participant groups (WK: *F* = 7.9, *p* = 0.001, Cx: *F* = 6.9, *p* = 0.003, rep-Cx: *F* = 7.1, *p* = 0.003). However, on paired comparisons, there was no difference in T1 results between HV and HF cohorts, while mean cortical T1 values were 122.4 ms (*p* = 0.009) and 84.7 ms (*p* = 0.02) greater in the Tx group compared to HV and HF groups, respectively. Medullary T1 values were also higher in Tx than HV (mean difference 129.1 ms, *p* = 0.03). There were no differences between groups on any cortical ROI for pCASL or ADC. Medullary pCASL values were significantly lower in Tx group compared to HV (mean difference − 35.7 ml/min/100 g, *p* = 0.03) and HF (mean difference − 48.4 ml/min/100 g, *p* = 0.03).

### Correlation between renal MRI and kidney function

eGFR data were available for the 30 participants with heart failure or a renal transplant. There was no correlation between eGFR and renal volume, T1 or pCASL. There was a positive correlation between eGFR and ADC (Tx group only), with coefficients of: WK 0.47 (*p* = 0.04), Cx 0.61 (*p* = 0.006), rep-Cx 0.72 (*p* = 0.001), sup-Cx 0.45 (*p* = 0.05), inf-Cx 0.67 (*p* = 0.002) and Med 0.48 (*p* = 0.04).

## Discussion

This study provides evidence to support the reproducibility of certain analysis techniques for renal MRI using commercially available analysis software. This is an essential step to allow studies exploring the clinical significance of functional renal MRI to report in confidence. Our data show that measurement of renal volume by contouring a localiser image is highly reproducible between observers. Contouring alternate slices, as opposed to every slice, results in a small reduction in measured volume with the advantage of improved efficiency. We believe the 1.6 ml (0.8%) mean difference, in volume by contouring alternate slices, is clinically insignificant, but nevertheless we would advise consistency with whichever approach is chosen. Whilst automated contouring and volume calculation is being utilised by some centres [16] and is likely to improve time efficiency, this approach is still to be externally validated and widely available. For T1, pCASL and ADC, WK ROIs are highly reproducible and commonly reported, but the mean value is derived from an unduly wide range of values, as evidenced by the fact on average the ROI SD represented between 17 and 55% of the mean value in our cohort. We would argue this summary statistic is a crude representation of the physiological tissue, which we hope to describe and that cortical values may have more biological relevance, without unacceptable reduction in reproducibility. Indeed, for ADC, the correlation with renal function of cortical ROIs was stronger than for WK. When drawing a small ROI of representative cortex, pre-specifying its location to be at either the superior (sup-Cx) or inferior (inf-Cx) pole did not improve reproducibility compared to a user-defined location and reduced the correlation with total cortex (Cx) for T1 and pCASL. Furthermore, sup-Cx and inf-Cx are theoretically more susceptible to artefact from respiratory movement in native kidneys compared to regions of lateral/medial cortex that would move in plane. We therefore advise that either Cx or rep-Cx be used preferentially, whenever cortical values are reported. Drawing an ROI for rep-Cx is likely to reduce analysis time compared to whole cortex and in this small sample, the correlation between eGFR and ADC was greatest when rep-Cx was used. However, this is balanced against the lower ICC for rep-CX than Cx. Further studies are required to distinguish their benefits and we suggest that either Cx or rep-Cx can be used to report cortical values in the interim. Nevertheless, development of a harmonised approach across centres is vital to allow broader use of renal MRI in research and clinical settings [1].

While there was a significant correlation between ADC and eGFR, there was no association between renal volume, T1 and pCASL with renal function. Although this may generate scepticism with regards to the clinical relevance of these sequences, the development of MRI biomarkers is intended to provide physiologic and prognostic information additional to existing clinical measures, but further studies are needed to clarify this.

We performed a limited comparison of medullary values. Future studies may wish to analyse the medulla in more detail. Recent studies have reported measures of corticomedullary differentiation (CMD) using T1 and ADC and their correlation with clinical parameters [17–19]. These studies were well-conducted, but there is a risk of over interpreting the significance of cortico-medullary findings. Loss of CMD is a well-established, non-specific finding in CKD that is detectable on ultrasound, computed tomography and MRI [20]. Any observed association between eGFR and CMD on T1 or ADC may underplay the utility of MRI as a functional measurement and may instead detect a crude structural change that is prevalent in CKD, and which can be measured in simpler ways.

The study is strengthened by its multi-parametric protocol across both healthy and diseased populations, including native and transplant kidneys yielding clinically meaningful results. The study has a number of limitations. Whilst we have shown these analyses to be reproducible, the clinical significance of any approach is not yet established. We did not assess R2* [also known as blood-oxygen-level-dependent (BOLD) imaging]. This parameter is recommended to be included in multi-parametric renal MRI protocols and its inclusion in this study would have been advantageous [1]. Only two observers reported each ROI for comparison of interobserver reproducibility. Kidney volume measurements were not compared with established 3D contrast-enhanced techniques, and further studies are required to assess the clinical relevance of kidney volume as measured by this approach. The current pCASL sequences utilise a fixed T1 value. We accept there may be advantages of using a measured T1 and we are exploring this for future studies. Other centres have developed efficient and accurate analysis methods, often using in-house developed software, which we are unable to replicate. For instance, a technique that uses a histogram to numerically segregate cortical from medullary values has been reported [4]. These analysis strategies require bespoke software which generally relies upon precise harmonisation of acquisition parameters to allow use out-with the centre in which they are developed. Nevertheless, comparison of results generated using this technique with the approaches detailed here would be interesting. The use of commercially available software in this study is strength. However, the license carries a cost and the software used is designed for cardiovascular analysis, such that we have applied many of the modules out-with their intended use. There is an urgent need for widely available software that is specifically designed for multi-parametric renal MRI analysis to advance the research and clinical application of renal MRI.

## Conclusion

There are numerous strategies to analyse multi-parametric renal MRI with many centres using in-house bespoke software. The optimal approach is not yet known. These results provide justification for one approach using commercially available software. We suggest that kidney volume can be calculated by contouring alternate slices, rather than every slice, of a localiser scan albeit validation with 3D volume techniques is still required. For T1, pCASL and ADC, we suggest that whole kidney values, while highly reproducible, are used with caution given that the results represent a central value from an extremely wide range. Instead, manually delineated cortex or a small ROI of user-defined representative cortex can be used interchangeably in both native and transplant kidneys, with acceptable interobserver reproducibility. Clinical correlation of the results generated from this approach is eagerly awaited.

## Electronic supplementary material

Below is the link to the electronic supplementary material.

Supplementary material 1 (DOCX 532 kb)
