# Supplementary material for: Comparing the interobserver reproducibility of different regions of interest on multi-parametric renal magnetic resonance imaging in healthy volunteers, patients with heart failure and renal transplant recipients
Source: MAGMA. 2019 Dec 10;33(1):103–12. doi: 10.1007/s10334-019-00809-4 (PMC7021749; doi:10.1007/s10334-019-00809-4)

**Supplementary material**

Supplementary material table 1. Imaging acquisition parameters for renal transplant and healthy volunteer cohorts

|  | **Volume** | **T1 map** | **pCASL** | **DWI** |
| --- | --- | --- | --- | --- |
| **Orientation** | Coronal | Coronal oblique | Coronal oblique | Coronal oblique |
| **Sequence** | TrueFISP* | MOLLI | pCASL with 3D TGSE readout | 2D SE-EPI |
| **TR (ms)** | 417 | Reported TR 550, echo spacing 3.04 | 6870 | 2300 |
| **TE (ms)** | 1.31 | 1.24 | 30.2 | 45 |
| **Flip angle (°)** | 37 | 35 | 28 (pCASL labelling)  180 (TGSE readout) | 90 |
| **Field of view (mm*mm)** | 340×340 | 360*215 | 300*150 | 400*400 |
| **Matrix** | 169*256 | 320*252 | 96*48 | 134*134 |
| **Slice thickness (mm)** | 4.5 | 5 | 4 | 5 |
| **Slice gap (mm)** | -1.125 | - | - | 1 |
| **Voxel size (mm^3^)** | 1.3*1.3*4.5 | 1.1*1.1*5 | 3.3*3.3*4 | 1.5*1.5*5 |
| **Number of slices** | 70 | 1 | 16 | 17 |
| **Acceleration** | GRAPPA R=2 | GRAPPA R=2, phase partial Fourier 7/8 | - | GRAPPA R=3 |
| **Acquisition time (min:sec)** | 00:35 | 00:10 | 03:33 (15 measurements) | 01:40 |
| **Bandwidth (Hz/px)** | 850 | 1116 | 2265 | 2488 |

*For TrueFISP acquisition, a Fat-Sat method, where fat appears nulled rather than bright, was applied to improve localisation of transplant kidneys. This protocol was applied to healthy volunteers.

Supplementary material table 2. Imaging acquisition parameters for heart failure cohort

|  | **Volume** | **T1 map** | **pCASL** |
| --- | --- | --- | --- |
| **Orientation** | Coronal | Coronal oblique | Coronal oblique |
| **Sequence** | TrueFISP | MOLLI | pCASL with 3D TGSE readout |
| **TR (ms)** | 553 | Reported TR 295, echo spacing 2.44 | 6870 |
| **TE (ms)** | 1.68 | 1.12 | 31.22 |
| **Flip angle (°)** | 50 | 35 | 28 (pCASL labelling)  180 (TGSE readout) |
| **Field of view (mm*mm)** | 340*380 | 360*307 | 150*300 |
| **Matrix** | 460*512 | 256*169 | 48*96 |
| **Slice thickness (mm)** | 5 | 8 | 4 |
| **Slice gap (mm)** | 0 | - | - |
| **Voxel size (mm^3^)** | 0.7*0.7*5.0 | 1.4*1.4*8 | 3.1*3.1*4 |
| **Number of slices** | 39 | 1 | 16 |
| **Acceleration** | GRAPPA R=2 | GRAPPA R=2, phase partial Fourier 7/8 | - |
| **Acquisition time (min:sec)** | 00:51 | 00:10 | 2:24 (10 measurements) |
| **Bandwidth (Hz/px)** | 1500 | 1085 | 2265 |

Supplementary material figure 1: Coronal, transverse and sagittal views showing the positioning of the ASL labelling plane (yellow) and the imaging volume (green) on a patient in the heart failure cohort.


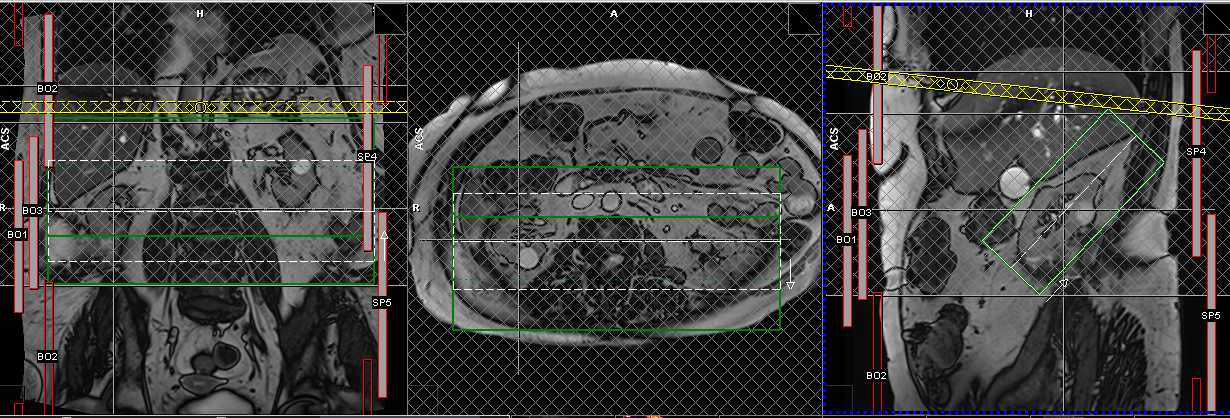

Supplement: Supplementary file 1 — Supplementary material 1 (DOCX 532 kb) [file 10334_2019_809_MOESM1_ESM.docx]
